# Potential Mechanisms Underlying the Dysfunction of the Blood–Brain Barrier

**DOI:** 10.3390/ijms24098184

**Published:** 2023-05-03

**Authors:** Julie E. Simpson

**Affiliations:** Sheffield Institute for Translational Neuroscience, The University of Sheffield, Sheffield S10 2HQ, UK; julie.simpson@sheffield.ac.uk

The blood–brain barrier (BBB) comprises a highly specialised complex of cells within the neurovascular unit, and is responsible for tightly regulating homeostasis within the central nervous system, which is critical for maintaining neuronal function. A loss of BBB integrity can lead to pathological permeability, resulting in the entry of immune cells and/or molecules into the central nervous system. This can stimulate a neuroinflammatory response, as well as ion dysregulation and altered signalling homeostasis, processes that contribute to neuronal dysfunction and neurodegeneration.

This Special Issue provides a comprehensive synopsis of current BBB research, taking in topics ranging from cellular to molecular mechanisms, the relationship between BBB dysfunction and the neuroinflammatory response, and cell models to study the molecular mechanisms underlying the pathophysiology of BBB disruption. Lindenau et al. consider the impact of Omega-3 polyunsaturated fatty acid dietary supplements on the BBB and demonstrate that the activation of free fatty acid receptor 1 produces a transient increase in permeability both in vivo and in vitro [1]. Vázquez-Villaseñor et al. performed an RNAseq analysis of neutrophil-derived microvesicles and identified a panel of miRNAs that may alter the integrity of the BBB and contribute to the accelerated cognitive decline seen as a result of systemic infection in Alzheimer’s Disease (AD) patients [2]. Using brain-like endothelial cells co-cultured with pericytes, Versele et al. identified potential mechanisms whereby pro-inflammatory cytokines associated with the neuroinflammatory response differentially induce changes in permeability and transcelluar trafficking in their model, as well as decreasing the efflux of Aβ peptide [3]. Deciphering the mechanisms underlying the dysfunction of the BBB is an important area for identifying and developing potential disease-modifying treatments in AD.

## Data Availability

Not applicable.
